# Obesity phenotypes related to musculoskeletal disorders; a cross-sectional study from RaNCD cohort

**DOI:** 10.1186/s13690-022-00947-7

**Published:** 2022-08-09

**Authors:** Sheno Karimi, Yahya Pasdar, Behrooz Hamzeh, Azad Ayenehpour, Fatemeh Heydarpour, Farjam Goudarzi

**Affiliations:** 1 Department of Nutritional Sciences, School of Nutritional Sciences and Food Technology, Kermanshah University of Medical Sciences, Kermanshah, Iran; 2 Research Center for Environmental Determinants of Health (RCEDH), Health Institute, Kermanshah University of Medical Sciences, Kermanshah, Iran; 3 Social Development and Health Promotion Research Center, Health Institute, Kermanshah University of Medical Sciences, Kermanshah, Iran; 4 Regenerative Medicine Research Center (RMRC), Kermanshah University of Medical Sciences, Kermanshah, Iran

**Keywords:** Metabolically unhealthy, Obesity, Low back pain, Low back stiffness, Arthralgia, Persian

## Abstract

**Background:**

This study was evaluated the association between obesity phenotypes and risk of lower torso musculoskeletal disorders including low back pain (LBP), low back stiffness (LBS), arthralgia, and joint stiffness in Ravansar non-communicable diseases (RaNCD) cohort study.

**Methods:**

In this cross-sectional study, 6940 adults were examined for the presence of lower torso musculoskeletal disorders by a physician. Obesity phenotypes including metabolically healthy obesity (MHO) and metabolically unhealthy obesity (MUO) were defined based on the International Diabetes Federation, as well as, body mass index > 30 kg/m^2^. Metabolically unhealthy non-obesity (MUNO) phenotype was considered as unhealthy metabolic without obesity.

**Results:**

The prevalence of LBP, LBS, arthralgia, and joint stiffness in MHO, MUO, and MUNO were significantly higher than in healthy participants compared to obesity phenotypes. Logistic regression showed that MHO phenotype was significantly increased with risk of LBP (OR: 1.19, CI 95%: 1.01–1.41), LBS (OR: 1.44, CI 95%: 1.12–1.86), arthralgia (OR: 1.54, CI 95%: 1.33–1.78), and joint stiffness (OR: 1.84, CI 95%: 1.35–2.52). Moreover, MUO phenotype was positively associated with risk of LBS (OR: 1.46, CI 95%: 1.09–1.94) and arthralgia (OR: 1.66, CI 95%: 1.41–1.96). In addition, MUNO phenotype was associated with a higher risk of arthralgia (OR: 1.21, CI 95%: 1.06–1.37).

**Conclusion:**

All three phenotypes, MHO, MUO and MUNO were significantly increased the risk of arthralgia. However, MHO phenotype was significantly associated with a higher risk of all examined lower torso musculoskeletal disorders in the current study.

## Background

Most lower torso musculoskeletal disorders are overlooked, perhaps because they are not directly related to mortality and are considered an irreversible process associated with aging. Although having these disorders increases treatment costs and reduces occupational and social activities, and overall, people with musculoskeletal disorders suffer to do their daily activities [1, 2]. Low back pain is the most common type of musculoskeletal disorder that people with this disease frequently refer to doctors for their treatment [3, 4]. Although disc herniation, lumbar stenosis, trauma, muscle strain, lumbar spondylosis, arthritis, spine and kidney infections, cancers, endometriosis, and ankylosing spondylitis are involved in its pathogenesis, low back pain without specific cause is the most prevalent [5]. Arthralgia is another musculoskeletal disorder that is a hallmark of rheumatoid arthritis [6]. Arthralgia is associated with joint pain and stiffness, and most patients complain of morning joint stiffness [7].

Due to healthy lifestyle changes, obesity is one of the serious health problems and has a high prevalence worldwide. Evidence suggests that obesity and a sedentary lifestyle increase comorbidities such as diabetes, cardiovascular disease, hypertension, and cancer, as well as, musculoskeletal pain [8–11]. However, obesity appears to increase the risk of these comorbidities by increasing inflammatory levels in the body, insulin resistance, and other metabolic disorders in which is often defined as metabolically unhealthy obesity (MUO) [9, 12]. While it has been seen that people, who are obese but are metabolically healthy are called metabolically healthy obese (MHO). On the other hand, metabolically unhealthy non-obese (MUNO) phenotypes have unhealthy metabolic profiles without obesity that are susceptible to to insulin resistance, dyslipidemia, and hypertension, and inflammatory-cardiometabolic abnormalities [13–15].

Many studies have examined the association between obesity and the risk of low back pain and arthralgia [9, 16]. However, to date, no study has examined the various phenotypes of obesity and metabolic profiles associated with the risk of low back pain and arthralgia. Therefore, this study aimed to determine the relationship between MHO and MUO phenotypes and the risk of back pain and arthralgia in the Kurdish population participated in the Ravansar non-communicable diseases (RaNCD) cohort study.

## Methods

### Study design and setting

We designed this cross-sectional study on baseline data of the RaNCD cohort study in which this is the first Kurdish population-based study on 10,047 Kurdish participants (4764 men and 5258 women) aged 35–65 years living in Ravansar city, Kermanshah province, Western ofIran. All participants in this study completed an interviewer-administered questionnaire. The RaNCD is a subset of the PERSIAN (Prospective Epidemiological Research Studies in Iran) mega cohort study that was approved by the Ethics Committees in the Ministry of Health and Medical Education, the Digestive Diseases Research Institute, Tehran University of Medical Sciences, Iran. The details of this study were described in previous studies [17, 18]. The RaNCD cohort study was approved by the Ethics Committee of Kermanshah University of Medical Sciences (No: KUMS.REC.1394.318).

### Participants

**Fig. 1:**
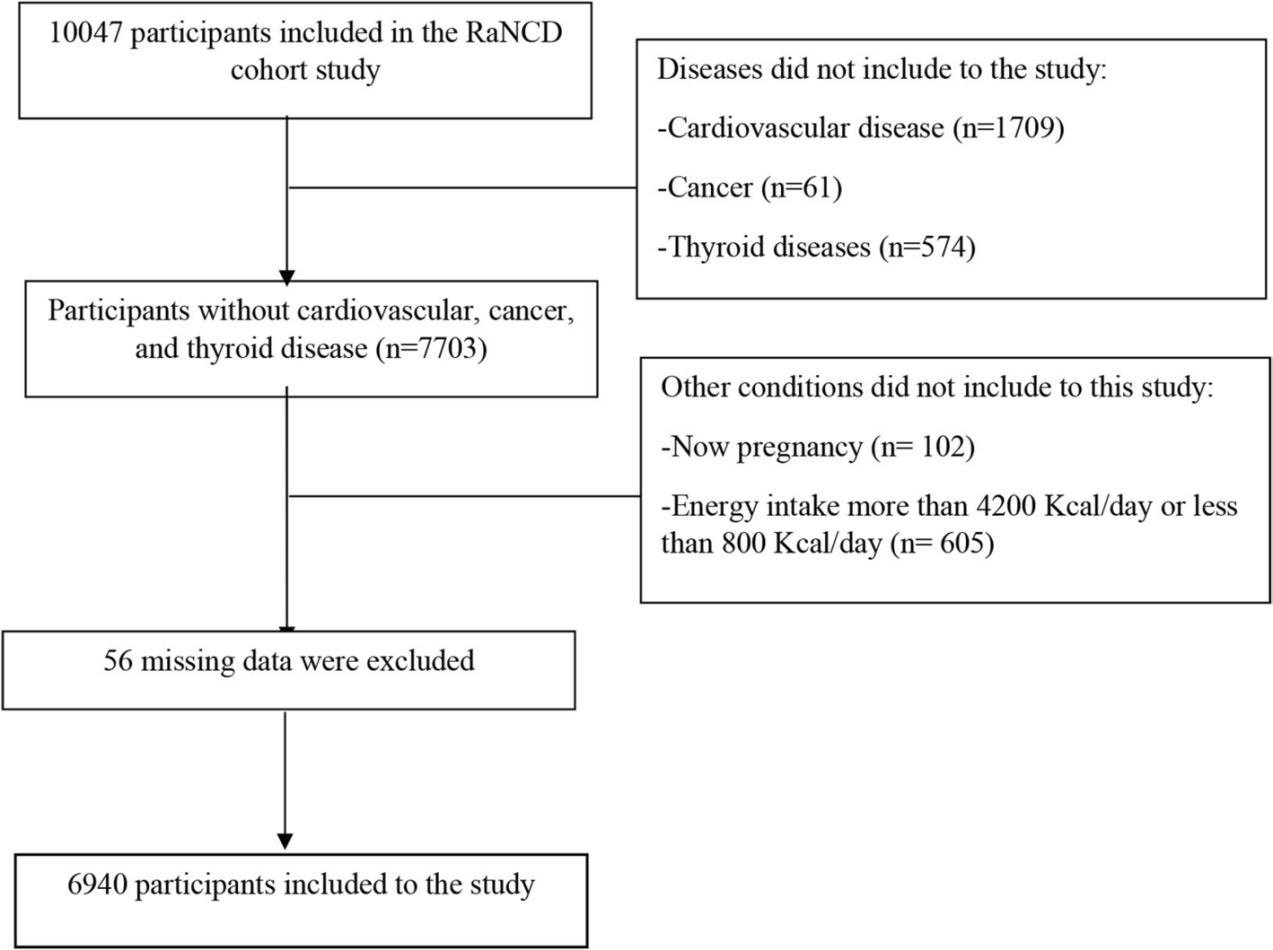
Flow chart of the sample selection

The current study did not include participants with cardiovascular diseases, cancer, and thyroid diseases. Besides, pregnant women and participants who were intake energy less than 800 kcal/day and more than 4200 kcal/day did not include in this study. Twins were excluded from analyses if: with a definitive diagnosis of alimentary tract tumor, cardiovascular heart disease, stroke and kidney disease; using weight-loss drug in the last month. After excluding participants with missing data, overall, 6940 participants were included in the study (Fig. 1).

### Anthropometry and body composition

In the RaNCD cohort study, height was measured using the automatic stadiometer BSM 370 (Biospace Co., Seoul, Korea) in the standing position without shoes with a precision of 0.1 cm. The body composition of the participants was measured by bioelectrical impedance analysis (InBody 770 device, Inbody Co, Seoul, Korea) including weight, body fat mass (BFM), and free fat mass (FFM) with the least clothing and without shoes. To measure height, Body mass index (BMI) was calculated by dividing weight (kg) by body height (m) squared. To measure waist circumference (WC) non-stretched and flexible tape was applied in a standing position at the level of the iliac crest.

### Blood pressure

The participant's systolic and diastolic blood pressure (SBP and DBP) was measured by conventional sphygmomanometry and auscultation of the Korotkoff sounds after at least 4–5 min of rest in sitting position from both arms of all participants two times with ten minutes interval between two times measurements and the mean of them was recorded as the final blood pressure [17].

### Biochemical analysis

In the RaNCD cohort study, all participants were obtained 25 cc venous blood samples. After separating serum, serum and whole blood samples were stored at -80^◦^C in the RaNCD cohort laboratory until analysis. The glucose oxidase method was applied to measure serum fasting blood sugar (FBS). Total cholesterol (TC), high-density lipoproteins (HDL), triglyceride (TG), and low-density lipoproteins (LDL) concentration were measured by enzymatic kits (Pars Azmun, Iran) [17].

### Obesity phenotypes

We considered MHO participants with BMI > 30 kg/m^2^ with healthy metabolic profiles based on the metabolic syndrome definition of the International Diabetes Federation (IDF) [19]. The MUNO has defined the presence of at least two metabolic disorder based on the IDF [19] criteria as follow:

-HDL < 40 mg/dl in men and < 50 mg/dl in women.
- Increased TG > 150 mg/dl.
- SBP > 130 mmHg or DBP > 80 mmHg.
- FBS > 100 mg/dl.

Also, metabolically unhealthy obesity (MUO) participants were considered participants having both obesity and the presence of at least two above metabolic disorder, as well as, metabolically healthy non-obesity (MHNO) participants were related to healthy participants without obesity and metabolic disorder.

### Outcome measurement

The RaNCD cohort study physician diagnosed the low back pain and arthralgia by medical history, lower torso musculoskeletal disorders self-report, and physical examination. Besides, all participants were surveyed in terms of the history of low back stiffness and joint stiffness by the physician using these questions 1) Do you experience low back pain that lasted more than a few months and interfered with their daily activities? In addition, has it lasted so far? (Yes/ No); 2) Do you have a history of back stiffness for more than an hour in the morning? (Yes/ No); 3) Do you have a history of arthralgia? (Yes/ No); 4) Do you have a history of joint stiffness for more than an hour in the morning? (Yes/ No). These questions and method of chronic diseases diagnosis were developed by the PERSIAN mega cohort study for all Iranian adults [20]. Furthermore, these patients with low back pain and arthralgia were specifically examined in terms of the presence of malignancies, infections, and fractures that the low back pain related to mentioned disorders was not considered the low back pain.

### Statistical analysis

Stata, version 14 (Stata Corp, College Station, TX) was used for statistical analysis. The results of participants’ baseline characteristics were expressed as mean ± standard deviation and number of participants (percentage) and were compared using Chi-square and one way ANOVA test between obesity phenotypes. Logistic regression was used to determine the association between obesity phenotypes (MHNO, MHO, MUNO, and MUO) and lower torso musculoskeletal disorders (low back pain and stiffness, arthralgia, and joint stiffness) by odds ratios (OR) and confidence interval 95% (CI 95%) for binary outcomes in crude and adjusted models (stepwise). *P*-values were considered significant at the level of *P* < 0.05.

## Results

In the current study, 51% of the studied participants were male. The prevalence of low back and joint stiffness, and arthralgia of MHNO participants were significantly lower than the other three studied groups (*P* = 0.002, < 0.001, 0.002, respectively).

The mean of MHNO physical activity was 42.06 ± 8.88 in which was significantly higher than the other three studied groups (*P* < 0.001). The mean of BFM and WC in MHO, MUO, and MUNO phenotypes were significantly higher than MHNO phenotypes (*P* < 0.001). The basic characteristics of the studied participants are presented in Table 1.

**Table 1:** Baseline characteristics, anthropometric and biochemical of participant’s factors according to the obesity phenotypes

Variables	Total (*n* = 6940)	MHNO (*n* = 3825)	MHO (*n* = 963)	MUNO (*n* = 1467)	MUO (*n* = 690)	*P* value**
**Age (year)**	46.19 ± 7.89^a^	45.83 ± 8.06	44.93 ± 7.05	47.63 ± 8.00	46.88 ± 7.35	< 0.001
**Sex, male (%)**	51	60.4	7.1	25	7.4	< 0.001
**Weight (kg)**	72.46 ± 13.58	66.75 ± 11.12	84.99 ± 11.03	71.97 ± 10.10	87.51 ± 12.57	< 0.001
**WC (cm)**	96.51 ± 10.35	92.08 ± 8.65	106.95 ± 8.28	96.00 ± 7.16	107.59 ± 8.60	< 0.001
**BMI (kg/m**^**2**^**)**	27.10 ± 4.69	24.73 ± 3.32	33.13 ± 2.95	26.40 ± 2.64	33.38 ± 3.31	< 0.001
**BFM (kg)**	24.28 ± 9.42	19.76 ± 6.62	36.39 ± 6.79	22.42 ± 5.25	36.24 ± 7.61	< 0.001
**FFM (kg)**	48.16 ± 9.40	47.00 ± 9.14	48.49 ± 9.10	49.56 ± 9.30	51.13 ± 10.34	< 0.001
**SBP (mmHg)**	105.88 ± 15.23	102.51 ± 12.95	105.12 ± 12.30	111.30 ± 17.44	114.16 ± 18.85	< 0.001
**DBP (mmHg)**	68.76 ± 9.30	66.85 ± 7.94	68.49 ± 8.23	71.70 ± 10.62	73.49 ± 11.24	< 0.001
**FBS (mg/dl)**	95.05 ± 26.88	89.92 ± 20.63	90.59 ± 15.22	105.68 ± 37.22	107.12 ± 33.01	< 0.001
**TC (mg/dl)**	184.92 ± 37.20	180.25 ± 37.08	186.93 ± 32.47	191.45 ± 38.47	194.12 ± 37.36	< 0.001
**TG (mg/dl)**	134.98 ± 81.64	101.88 ± 46.50	114.22 ± 47.21	205.78 ± 102.08	196.83 ± 93.27	< 0.001
**HDL (mg/dl)**	46.44 ± 11.38	49.65 ± 11.18	49.96 ± 10.59	38.72 ± 8.17	40.18 ± 8.55	< 0.001
**LDL (mg/dl)**	101.81 ± 25.06	98.82 ± 24.99	102.89 ± 22.18	106.12 ± 25.93	107.78 ± 24.88	< 0.001
**PA (MET hour/ week)**	41.22 ± 8.32	42.06 ± 8.88	39.79 ± 6.33	40.86 ± 8.31	39.36 ± 6.83	< 0.001
**Low back pain (%)**	21.9	21.2	24.4	21.9	22.2	0.208
**Low back stiffness (%)**	7.2	6.3	9.3	6.6	9.4	0.002
**Arthralgia (%)**	34.9	31.4	41.4	35.7	43.3	< 0.001
**Joint stiffness (%)**	4.2	3.5	6.3	4.2	4.3	0.002

^a^Mean ± SD

^**^P-values were obtained one-way ANOVA and Chi-square

Multivariable-adjusted odds ratios and 95% confidence intervals for lower torso musculoskeletal disorders across categories of metabolically status were showed there was a significant association between low back pain and low back stiffness with MHO phenotype compared to MHNO phenotype (OR: 1.19, CI 95%: 1.01–1.41), (OR: 1.44, CI 95%: 1.12–1.86), respectively in crude model. After adjusted for age, gender, education, and physical activity, any association was not observed (OR: 1.17, CI 95%: 0.98–1.39), (OR: 1.24, CI 95%: 0.95–1.6). Moreover, MHO phenotype was significantly increased with risk of arthralgia, and joint stiffness in all studied models (Table 2).

**Table 2:** Multivariable-adjusted odds ratios and 95% confidence intervals for lower torso musculoskeletal disorders across categories of obesity phenotypes

**Musculoskeletal disorders**		**MHNO** **(*n* = 3825)**	**MHO** **(*n* = 963)**	**MUNO** **(*n* = 1467)**	**MUO** **(*n* = 690)**	**P-trend**
**Low back pain**	Crude	1	1.19 (1.01–1.41) ^a^	1.04 (0.9–1.2)	1.05 (0.86–1.28)	0.431
	Model 1^b^	1	1.17 (0.98–1.39)	1.01 (0.87–1.18)	1.01 (0.83–1.23)	0.737
	Model 2 ^c^	1	1.17 (0.98–1.39)	1.02 (0.88–1.18)	1.01 (0.83–1.23)	0.719
**Low back stiffness**	Crude	1	1.44 (1.12–1.86)	0.95 (0.75–1.21)	1.46 (1.09–1.94)	0.086
	Model 1	1	1.23 (0.95–1.59)	0.95 (0.74–1.22)	1.3 (0.97–1.73)	0.249
	Model 2	1	1.24 (0.95–1.6)	0.96 (0.75–1.24)	1.31 (0.98–1.75)	0.221
**Arthralgia**	Crude	1	1.54 (1.33–1.78)	1.21 (1.06–1.37)	1.66 (1.41–1.96)	< 0.001
	Model 1	1	1.36 (1.17–1.58)	1.17 (1.02–1.33)	1.47 (1.24–1.74)	< 0.001
	Model 2	1	1.36 (1.17–1.59)	1.17 (1.03–1.34)	1.48 (1.25–1.75)	< 0.001
**Joint stiffness**	Crude	1	1.84 (1.35–2.52)	1.2 (0.89–1.63)	1.24 (0.83–1.86)	0.121
	Model 1	1	1.55 (1.13–2.14)	1.16 (0.85–1.59)	1.05 (0.7–1.59)	0.418
	Model 2	1	1.55 (1.12–2.13)	1.17 (0.85–1.59)	1.05 (0.69–1.58)	0.435

*MHNO *metabolically healthy non-obese, *MHO *metabolically healthy obese, *MUNO *metabolically unhealthy non-obese, *MUO *metabolically unhealthy obese

^a^OR (CI 95%),

^b^Model 1 adjusted for age and gender

^c^Model 2 adjusted for confounder variables in Model 1, education, and physical activity

**Fig. 2:**
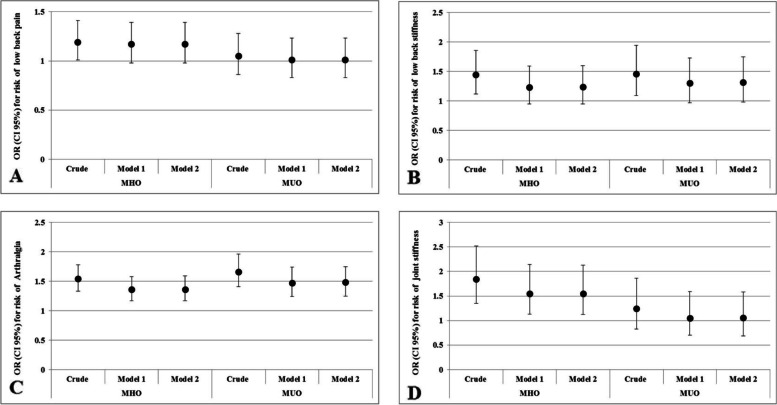
Binary regression odds ratios and 95% confidence intervals for **A** Low back pain; **B** Low back stiffness; **C** Arthralgia, and **D** Joint stiffness across categories of obesity phenotypes. MHNO: metabolically healthy non-obese; MHO: metabolically healthy obese; MUNO: metabolically unhealthy non-obese; MUO: metabolically unhealthy obese

We also observed MUO phenotype was positively associated with risk of low back stiffness (OR: 1.46, CI 95%: 1.09–1.94) in crude model. After adjusted for mentioned confounder variables, there was no association between low back stiffness and MUO (OR: 1.31, CI 95%: 0.95–1.75) (Table 2). Furthermore, MUNO phenotype was associated with a higher risk of arthralgia (OR: 1.17, CI 95%: 1.03–1.34). Figure 2 showing a better manifestation of above association of musculoskeletal disorders (low back pain and stiffness, arthralgia and joint stiffness) by binary regression OR across categories of MHO and MUO phenotypes.

## Discussion

We found that the prevalence of all types of lower torso musculoskeletal disorders examined in the current study in MHO, MUO, and MUNO phenotypes was significantly higher than the MHNO phenotype. In this study, all three examined phenotypes, MHO, MUO, and MUNO lead to increased risk of arthralgia. Overall, MHO was related to a higher risk of all types of lower torso musculoskeletal disorders examined in this study compared to MHNO, as well as, MUO was associated with a higher risk of low back stiffness. Recent studies have highlighted that obesity increases the risk of musculoskeletal pain in the lower torso [16, 21]. Therefore, according to the phenotypes of obesity based on metabolic status, it seems that the present study can help to identify one of the factors affecting the reduction of the risk of musculoskeletal pain in the lower torso.

This present study showed that the MHO phenotypes were related to the increased risk of all types of lower torso musculoskeletal disorders. MHO might be an alternative valuable target in obesity treatment. In a cross-sectional analysis using the China Multi-Ethnic Cohort study that enrolled 99 556 participants from seven diverse ethnic groups, marginal structural logistic models were used to estimate the associations. Among 65,699 participants, 11% were with obesity. MHO phenotype was present in 5·7% of total population and 52·7% of population with obesity. Results showed that Dietary Approaches to Stop Hypertension (DASH) diet were associated positively with obesity and MHO phenotype [22].

Also, MUO phenotypes were associated with a higher risk of low back stiffness and arthralgia. Blumel et al. [23] in a study on women aged 40–59 years observed that obesity increased the risk of musculoskeletal pain (OR: 1.34; CI 95%: 1.16–1.55). Results from a systematic review and meta-analysis by Walsh et al. [16] on 14 studies showed that obesity is associated with increased pain in the joints, back, legs and knees due to the increase in body fat tissue.

Problems with the physical aspect of the elderly, one of which is a decrease in musculoskeletal function, especially in the joints, often manifest the joint pain. The presence of joint cartilage damage due to inflammation, as well as an increase in the load on the joints due to obesity is other factors that often arise. Masruroh and Setyadhani showed a direct correlation between obesity level and the incidence of knee joint pain in the elderly in a cross-sectional approach on 50 individuals [24]. A proposed mechanism associated with obesity and musculoskeletal disorders arises from the production of pro-inflammatory cytokines from high excess adipose tissue in obese individuals [25]. Leptin is a pro-inflammatory adipokine that is highly elevated in obese individuals in which leads to deforming cartilage and be involved in the pathogenesis of arthralgia [26, 27]. Besides, leptin with inflammatory properties can lead to degeneration of the intervertebral discs and consequently low back pain [27]. Other important inflammatory cytokines involved in increasing joint inflammation and musculoskeletal pain are related to increased levels of tumor necrosis factor-alpha (TNF-α), interleukin 6, and C- reactive protein (CRP) [26, 28, 29]. The production of these cytokines has been seen in obese people with increased adipose tissue [30]. According to the findings of the present study, BFM and WC in MHO and MUO phenotypes are significantly higher than MHNO phenotype and can indicate the cause of increased risk of lower torso musculoskeletal disorders in both mentioned phenotypes compared to MHNO. In a cross-sectional study, the different subtypes of obesity and their relationship with inflammatory-cardiometabolic abnormalities was investigated in Chinese adult twins. Results showed that MHO and MUNO phenotypes were common in Chinese twin population. Both phenotypes were associated with elevated insulin resistance and high sensitivity C reactive protein (hsCRP) which may not be benign and need to be concerned [15]. Additionally, we observed that MUNO phenotypes were associated with a higher risk of arthralgia compared to MHNO phenotypes. Although these participants were not obese, according to the high BMI, WC, and BFM compared to MHNO phenotypes, they seem overweight, and increased adipose tissue seems to contribute to a higher risk of lower torso musculoskeletal disorders in this phenotype due to the mechanisms mentioned. Walrabenstein et al. designed three 16-week observer-blind randomized clinical trials (RCTs) with a waiting-list control group for patients with RA with low to moderate disease activity (2.6 ≤ Disease Activity Score [DAS28] ≤ 5.1, RCT 1, *n* = 80), for patients at risk for RA, defined by anti-citrullinated protein antibody (ACPA)-positive arthralgia (RCT 2, *n* = 16) and for patients with metabolic syndrome and osteoarthritis in the knee and/or hip (RCT 3, *n* = 80). Participants join 10 group meetings with 6–12 other patients to receive theoretical and practical training on a WFPD, exercise, and stress management, while medication remains unchanged. Primary outcomes were following difference in mean change between intervention and control groups within 16 weeks for the DAS28 in RA patients (RCT 1), the RA-risk score for ACPA positive arthralgia patients (RCT 2), and the Western Ontario and McMaster Universities Arthritis Index (WOMAC) score for MSOA patients (RCT 3) [31]. In a study on rheumatoid arthritis (RA) and spondyloarthritis (SpA) consuming disease-modifying anti-rheumatic drugs (DMARDs), the prevalence and correlation of metabolic syndrome and body mass index (BMI) were examined. In non-obese SpA, metabolic syndrome was associated with abdominal obesity, visceral fat mass and cardiovascular risk. In non-obese RA patients with metabolic syndrome, body composition did not differ from metabolically healthy RA patients. This Differences between RA and SpA for metabolic health suggest various pathophysiological mechanisms [32].

This present study for the first time evaluated the relationship between the obesity phenotypes and risk of lower torso musculoskeletal disorders in large Kurdish population- based study. Unfortunately, this study suffered from several limitations, including the degree and severity of each of these pain in the cohort study were not reported. Also, the cross-sectional design of our study should not be ignored; hence, these results seem should be supported by prospective follow-up well design studies.

## Conclusion

As a result, the findings of this study indicate the effect of both obesity phenotypes on the increased risk of lower torso musculoskeletal disorders. Therefore, weight control and management are an important strategy in reducing these disorders.

## Data Availability

The datasets generated during and/or analyzed during the current study are not publicly available due to the policy of the Persian cohort committee available at http://persiancohort.com/central-committee/ but are available from the corresponding author on reasonable request.

## Abbreviations

MHNO: Metabolically healthy non-obese
MHO: Metabolically healthy obese
MUNO: Metabolically unhealthy non-obese
MUO: Metabolically unhealthy obese
WC: Waist circumference
BMI: Body mass index
BFM: Body fat mass
FFM: Free fat mass
SBP: Systolic blood pressure
DBP: Diastolic blood pressure
FBS: Fasting blood sugar
TC: Total cholesterol
TG: Triglyceride
HDL: High density lipoprotein
LDL: Low density lipoprotein
PA: Physical activity
